# Internet-based search of randomised trials relevant to mental health originating in the Arab world

**DOI:** 10.1186/1471-244X-5-30

**Published:** 2005-07-26

**Authors:** Yahya Takriti, Hany G El-Sayeh, Clive E Adams

**Affiliations:** 1 Aire Court Community Unit, Lingwell Grove, Leeds, LS10 4BS, UK; 2 Academic Unit of Psychiatry and Behavioural Sciences, University of Leeds, 15 Hyde Terrace, Leeds, LS2 9LT, UK; 3 Cochrane Schizophrenia Group, Academic Unit of Psychiatry and Behavioural Sciences, University of Leeds, 15 Hyde Terrace, Leeds, LS2 9LT, UK

## Abstract

**Background:**

The internet is becoming a widely used source of accessing medical research through various on-line databases. This instant access to information is of benefit to busy clinicians and service users around the world. The population of the Arab World is comparable to that of the United States, yet it is widely believed to have a greatly contrasting output of randomised controlled trials related to mental health. This study was designed to investigate the existence of such research in the Arab World and also to investigate the availability of this research on-line.

**Methods:**

Survey of findings from three internet-based potential sources of randomised trials originating from the Arab world and relevant to mental health care.

**Results:**

A manual search of an Arabic online current contents service identified 3 studies, MEDLINE, EMBASE, and PsycINFO searches identified only 1 study, and a manual search of a specifically indexed, study-based mental health database, PsiTri, revealed 27 trials.

**Conclusion:**

There genuinely seem to be few trials from the Arab world and accessing these on-line was problematic. Replication of some studies that guide psychiatric/psychological practice in the Arab world would seem prudent.

## Background

The well-conducted randomised trial is the gold standard for the evaluation of medical interventions including mental health treatments [1]. This methodology was first formally used in the late 1940's [2] although much earlier examples do exist  including one controlled experiment from the Middle East recorded in the Bible about 2nd-1st century BC (Daniel 1:1–16). At first randomised trials were few in number and easy to summarise but the exponential rise of these studies made it increasingly difficult to produce clear unbiased summaries of the best evidence on the effects of health care interventions [3]. Currently there are thousands of journals worldwide which publish the results of trials and many thousands of randomised trials reported each year. Clinicians, trialists, policy makers and patients cannot hope to keep up with the annual volume of literature so reviews are useful.

Frequently, reviewing is subjective with little or no attempt to ensure that the results are reproducible and as free from bias as possible [3]. More systematic reviews, however, endeavour to minimise bias and combine the results of all relevant trials in objective, explicit and reproducible ways [4]. This is exemplified by the work of The Cochrane Collaboration, an international, independent and not-for-profit organisation which disseminates systematic reviews of healthcare interventions worldwide [5]. This Collaboration recognises that including only easily-identified trials in reviews leaves work vulnerable to the inclusion of bias, as highly accessible trials tend to be more positive than those that are more difficult to find [6-8]. Cochrane reviewers therefore make strenuous efforts to find all relevant studies-a model that all researchers would do well to emulate when searching for relevant randomised controlled trials.

The Arab world is a culturally, religiously and ethnically diverse region consisting of 22 countries from Mauritania in the west, to Iraq in the east. Its rapidly expanding population is estimated at over 280 million. The media in this region reports little, except in the context of oil reserves, religious tensions and conflict. Despite the Arab world having a population comparable in size to that of the USA, its contribution to recent global medical literature is thought to be relatively small [9]. Reasons for this are probably many and complex, but may include military conflict and arms expenditure, the 'brain drain', research culture, trade embargoes and humanitarian crises [9,10].

Although a comparatively small proportion of randomised controlled trials are conducted outside of the relatively affluent West, there is evidence that other parts of the developing world are beginning to make progress. One particular study looked at randomised controlled trials originating from Sub-Saharan Africa, and showed that there may have been an increase in publication of these trials over time. The same article however, identified that there was a relative lag between the overall burden of mental illnesses on the continent and trials focussing on these conditions [11].

This paper describes a search for, and survey of, randomised controlled trials relevant to mental health published in the Arab literature or having clearly originated from one of the countries of the Arab world.

## Methods

First we manually searched the ArabPsyNet database  The function of this internet-based database, in part, is as a current awareness service for the contents of Arab psychiatric and psychological periodicals. The list of journals and the years for which contents pages are available are presented in Table 1. An initial search was conducted in December 2003 and repeated in May 2004. Articles published in Arabic, English and French could have been included. We recognise that publication in Arab World journals did not necessarily imply the Arabic descent of authors and, similarly, publication in an Arab world journal did not necessarily mean that the research was undertaken in the Arab world.

**Table 1:** Number of issues available to search electronically in Arab psychiatric journals and number of randomised controlled trials identified. Table modified from

**Journal**	**Country of publication**	**Language**	**Dates available**	**Total number of. issues in available dates**	**No. issues with contents pages available on this site**	**No. RCTs identified on available content pages**
Addiction Bulletin	Egypt	Arabic	1999–2000	8	4	0
Arabpsynet journal	Tunisia	Arabic & English	2004–2004	2	2	0
Arab Journal of Psychiatry	Jordan	English	1989–2003	28	18	2
Arab Psychologist	Egypt	English	2000–2001	2	2	0
Assaha Al Aklia	Yemen	Arabic	1999–2001	12	9	0
Bulletin of Egyptian Psychiatric Association	Egypt	Arabic	1999–2001	12	5	0
Current Psychiatry	Egypt	English	1994–1996	6	3	1
Egyptian Journal of Mental Health	Egypt	Arabic & English	None	0	0	0
Egyptian Journal of Psychiatry	Egypt	English	None	0	0	0
Egyptian Journal of Psychological Studies	Egypt	Arabic	1992–2000	36	1	0
Interdisciplinary Psychology	Lebanon	Arabic	1990–2004	58	54	0
Journal on Arab Children	Kuwait	Arabic	1998–2004	24	13	0
Man and Evolution	Egypt	Arabic	1984–2000	76	41	0
Mental Health	Yemen	Arabic	1992–2000	18	7	0
Mental Peace Journal of WIAMH	Saudi Arabia	Arabic	1994–2002	36	12	0
News Letter of the AFNGO for Drug Abuse Prevention	Egypt	Arabic	1999–2001	6	2	0
Psychological Quarterly	Egypt	Arabic	2000–2002	12	4	0
Psychology	Egypt	Arabic	1987–2002	64	22	0
Tunisian Annals of Psychiatry	Tunisia	French	1996–1997	4	2	0
Tunisian Journal of Psychiatry	Tunisia	French	1998–2001	8	1	0
WIAMH Newsletter	Egypt	English	1998–2003	38	10	0

Next, we manually searched EMBASE, MEDLINE and PsycINFO with " (Randomi* and (arab* or [names of individual countries]) and ([broad terms for mental disorder/illness]))".

Thirdly, we manually searched PsiTri  for the name of every Arab country in the 'country of origin' field. PsiTri is a freely available based electronic database on published and unpublished controlled clinical trials, reporting on treatments and interventions for a wide range of conditions within the field of mental health. Uniquely, it is study-based, rather than simply consisting of lists of citations that could relate to the same study. It is also reliably indexed with country of origin.

## Results

The search of ArabPsyNet identified only three randomised controlled trials which had been published in the 212 available issues from the 21 journals between1984 and 2004. If all issues had been available from all 21 journals we predict that three more studies would have been identified. All three trials were published in English (see Table 1). The first, in the Arab Journal of Psychiatry in 1989, reports a small randomised controlled trial of alprazolam versus cognitive therapy for outpatients with panic disorders [12]. The second, published in 1996, also in the Arab Journal of Psychiatry [13] reports another small randomised controlled trial, this time comparing zuclopenthixol acetate with haloperidol for people with schizophrenic psychoses, affective psychoses and paranoid states. Finally, the third trial was published in Current Psychiatry in 1996 and reports a randomised controlled trial of sertaline versus placebo for over 300 people with obsessive-compulsive disorder [14]. This final trial was not conducted in the Middle East, but was published in an Egyptian journal.

MEDLINE and EMBASE searches did not identify any of the three studies. However, one of the studies [13] was identified through PsycINFO. The majority of studies included on these three popular databases come from journals with a high citation index, all of which tend to be published in the Western world. In this way, the low citation rate of Arab journals is likely to be perpetuated as these databases are commonly used in searches.

PsiTri contained most randomised controlled trials relevant to this survey. Searching on country of origin several relevant articles published outside of Arab journals, some of which were not in psychiatric or psychological periodicals. In all, we identified 27 randomised controlled trials from eight Arab countries (see Table 2).

**Table 2:** Results from PsyTri searching by country of origin

Country in which trial was thought to be undertaken	No. of randomised trials	Journal of publication	Subject	Year
Algeria	1	European Psychiatry	Depression [18]	1995
Bahrain	1	Arab Journal of Psychiatry	Psychoses [13]	1996
Egypt	12	Journal of International Medical	Depression [19]	1980
		Research	Depression [20]	1976
		Journal of International Medical	Impotence [21]	1992
		Research Journal of Urology	Affective disorders [22]	1983
		International Journal of Nursing	Depression [23]	1976
		Studies	Enuresis [24]	1999
		Journal of the Egyptian Medical	Enuresis [25]	1990
		Association	Pubertal	
		Urology	development [26]	1969
		Urology	Neurotic Disorders;	
		American Journal of Clinical	Schizophrenia;	
		Nutrition	Depressive	
		Acta Psychiatrica Scandinavica	Disorder; Mental Disorders [27]	1970
				
		Journal of the European College of Neuropsychopharmacology	Schizophrenia [28]	1999
		British Journal of Psychiatry	Schizophrenia [29]	1970
Iraq	2	Journal of Urology	Enuresis [30]	1989
		Clinical and Experimental Pharmacology & Physiology	Enuresis [31]	1986
Lebanon	2	Pediatric Nursing	Anxiety [32]	1998
		International Journal of Geriatric Psychiatry	Alzheimers [33]	2004
Libya	1	Gerontologia Clinica	Senile restlessness [34]	1970
Kuwait	3	British Journal of Psychiatry	ECT-induced cognitive	
		Journal of the Kuwait Medical	impairement [35]	1985
		Association	Schizophrenia [36]	1981
		Biological Psychiatry	Chronic schizophrenia [37]	1999
Saudi Arabia	5	Journal of Clinical	Depression [38]	1995
		Pharmacology	Anxiety [39]	1999
		British Journal of Anaesthesia	Dental anxiety [40]	1999
		Journal of Dental Research Acta Psyhiatrica Scandinivica	Psychiatric illness [41]	1997
		Behavioural and Cognitive Psychotherapy	Schizophrenia [42]	1997

## Discussion

ArabPsyNet, the only mental-health specific database detailing 'table of contents' pages of journals rarely seen outside of Arabic-speaking world, is making a concerted effort to disseminate research from the region. As yet the coverage of journals is limited and varied- as is the information produced on each article. As this site develops, its contribution should become greater. Current searches suggest that trials from the Arab world are rarely published in these journals. The main-stream databases are also not good sources of mental health trials from this region and may make the possibility of 'index-bias' more likely when searching for trial of Arab World origin. It is possible that we failed to identify studies, as reporting of country of origin is variable. Further complexity is added to these searches of general databases by having to use a phrase covering the many ways mental health problems are indexed, as well as one for the many country's names in title, abstract, and address fields. The mental health specific database, PsiTri, avoided the need for this. Being study-based and specifically indexed for country of origin confers great advantage over the other two sources. This database, as a compilation of all registers of mental health Cochrane groups (which are themselves created with extensive searches of many diverse databases) is likely to be the best source in existence of clearly indexed mental health studies from the Arab world.

It is possible that we failed to identify relevant trials published in Arab journals. Many countries, including The Czech Republic, Hungary, India, Korea, Latin America, Russia, and the Ukraine (see Table 3), recognising that coverage of their home literature in databases such as MEDLINE will always be limited, produce their own medical bibliographic databases in which searching for randomised trials is relatively simple. We know of no such database in the Arab world, but such a project would make a most valuable contribution to dissemination of medical research from the area.

**Table 3:** Bibliographic and full text sources by country/region of origin.*

AFRICA [43]
CHINA [44]
CZECH REPUBLIC [45]
EASTERN MEDITERRANEAN [46]
EGYPT [47]
FRANCE [48]
HUNGARY [49]
INDIA [50]
KOREA [51]
LATIN AMERICA [52]
POLAND [53]
RUSSIA [54] [55] [56]
SAUDI ARABIA [57]
THAILAND [58] [59] [60] [61]
UKRAINE [62]

* This list is not meant to be comprehensive – but is presented at request of peer review to illustrate the wealth of sources for bibliographic registers of biomedical literature

Lower profile Arab journals may not be able to attract submission of randomised trials from home or overseas. The results from PsiTri did suggest that studies from the Arab world are most often seen in journals outside of the region. There is evidence from other studies that trials from non-English speaking countries are more likely to be published in Anglophone journals if statistically significant and those same authors are more likely to publish their other, less 'significant' studies in their home language [15]. We cannot tell how many trials would have been sent to 'mainstream' journals and rejected and we would have expected to see them published in the home literature. We did not. With raised awareness amongst editors of publication bias due to study origins, we hope this phenomenon, if contributing to the dearth of accessible trials from the Arab world becomes less prevalent.

Perhaps there are many fewer studies published in the Arab world than would be expected from the population. Previous work suggests that population is not a good predictor of productivity of trials relevant to schizophrenia [16]. Gross Domestic Product (GDP) proved a much more potent predictor. The greater the GDP, the greater the productivity of schizophrenia trials. Assuming this holds for all mental health trials, one potent factor could be that many of the Arab countries are in the low income bracket where output of [at least] schizophrenia trials would be expected to be low. Even the few richer Arab countries have a GDP which is focused on oil revenue. This uneven poverty may well be a factor in the Arab world's lack of productivity of mental health trials. Although poverty may be predictive, it is not necessarily causative and this fact may not fully explain why trial numbers in other more economically-disadvantaged parts of the developing world appear to be increasing [11]. Not all trials are highly expensive. Infrastructure for trials, however, may be suffering from under investment, and the culture or political climate may be hostile. One cannot underestimate the potential effects that culture may play in clinical trial conduction in the region. These potential effects may be expected to be seen much more acutely in the much-stigmatised area of mental health. Many factors could mediate against evaluative research.

There were a few further areas related to the study on which the authors would like to make further comment on.

Although not specifically reported in this study mainly owing to poor data availability, it may have been useful to comment on the number of studies which reported statistically significant results- which in turn may give an indication of potential publication bias. Most of the included trials did not appear to focus on topics that had a particular Arab-focus and therefore, notwithstanding issues around inter-regional validity, results from large, well designed Western trials (albeit covering similar subject matters) may provide equally-accurate results on which to base local service provision. It was difficult to confirm these observations as we were only able to gain access to the table-of-contents pages for most of the electronic databases and hence had limited ability to comment on the regional nature of the research. Although our study looked only at randomised controlled trials on mental health, we acknowledge the possibility that valuable research in other methodological formats may still be published with an Arab-focus and may provide useful answers to health questions in the area. The author's also acknowledge that the overall quality of the reporting of studies may have been a useful additional measure, however it was seen as being outside of the remit of this article. The ethical nature of included studies again could have been reported on if further data have been available. There is some evidence that trials conducted in 'resource-poor settings' may be more likely to include comparator arms that would be deemed sub-optimal, or even unethical in the West [17]. Conversely, what is deemed ethical in the West could be unethical in situations where resources are not so abundant. Judging the ethics of others by standards that are not based in local knowledge and culture may be problematic.

There is also the issue of researchers migrating from the region. Researchers of Arab origin may leave to work in more wealthy countries where funding for randomised controlled trials is more readily available.

## Conclusion

Even if we have failed to identify many relevant studies, there is a suggestion that this large part of the world is not significantly contributing to evaluative mental health research. This perceived paucity of randomised controlled trials on mental health originating from the Middle East may in part be compounded by the lack of availability and dissemination of trial results via the internet. The apparent lack of randomised controlled trials in the Arab world could, in part, be due to a lack of funding. Despite the lack of randomised controlled trials, researchers in the Arab world are publishing studies on mental health issues. Although our research has highlighted the limited numbers of relevant trials accessible through the internet, it should be noted that there may be a substantial body of work being conducted and published in other formats. Creating a regional database may provide researchers with greater trial accessibility and hence locate a hitherto-'untapped well' of information.

## Competing interests

The author(s) declare that they have no competing interests.

## Authors' contributions

YT participated in the design of the study, carried out the literature search and joint drafted the manuscript. HES contributed to the analysis and interpretation of data and joint drafted the manuscript. CA participated in the design of the study, contributed to the analysis and interpretation of the data and revised the manuscript. All authors read and approved the final manuscript.

## Pre-publication history

The pre-publication history for this paper can be accessed here:


